# A review of common influencing factors and possible mechanisms associated with allergic diseases complicating tic disorders in children

**DOI:** 10.3389/fped.2024.1360420

**Published:** 2024-06-18

**Authors:** Panpan Zhang, Zhimin Zheng, Hao Sun, Tieying Gao, Xuwu Xiao

**Affiliations:** ^1^ Department of Child Health, Dalian Municipal Women and Children’s Medical Center (Group), Dalian, Liaoning, China; ^2^ Dalian Medical University, Dalian, Liaoning, China

**Keywords:** children, allergic diseases, tic disorders, common factors, mechanism

## Abstract

Over the past few decades, the incidence of childhood allergic diseases has increased globally, and their impact on the affected child extends beyond the allergy itself. There is evidence of an association between childhood allergic diseases and the development of neurological disorders. Several studies have shown a correlation between allergic diseases and tic disorders (TD), and allergic diseases may be an important risk factor for TD. Possible factors influencing the development of these disorders include neurotransmitter imbalance, maternal anxiety or depression, gut microbial disorders, sleep disturbances, maternal allergic status, exposure to tobacco, and environmental factors. Moreover, gut microbial disturbances, altered immunological profiles, and DNA methylation in patients with allergic diseases may be potential mechanisms contributing to the development of TD. An in-depth investigation of the relationship between allergic diseases and TD in children will be important for preventing and treating TD.

## 1 Introduction

Allergic diseases in children include food allergy (FA), atopic dermatitis (AD), allergic rhinitis (AR), allergic conjunctivitis (AC) and allergic asthma (AA), which are chronic inflammatory disease with symptoms such as hives, swelling, vomiting, diarrhea, itchy rashes on the skin, watery or itchy eyes, sneezing, congestion, tightness of chest coughing and reversible airflow limitation (1, 2). Allergic diseases are systemic disorders caused by an impaired immune system, and their pathogenesis is complex and involves many factors, including genetics, epigenetics, environmental factors, and the body's immune status (3). IgE is a key factor in the pathogenesis of allergic diseases. Allergens and IgE sequentially activate dendritic cells, and T and B cells to initiate allergic immune responses (4). Simultaneously, they activate mast cells, basophils, and eosinophils to release inflammatory mediators, resulting in allergic reactions (5). Not only do allergic diseases lead to a rise in school absenteeism and a decrease in children's involvement in outdoor activities (6), but studies have also suggested a possible link between these conditions and the development of neurological disorders (7), particularly Tic disorder (TD) (8).

TD is a group of chronic neurological disorders that begin in childhood and adolescence, usually occurring for the first time at the age of 5–6 years and is characterized by sudden, rapid, repetitive, stereotypical, non-rhythmic, non-rhythmic single- or multi-located muscular and/or vocal tics of an involuntary nature (9). TD can be categorized into Provisional tic disorders (PTD), Chronic motor or vocal tic disorders (CTD), and Tourette's syndrome (TS), depending on their characteristics and the progression of the disease (10). TD is often accompanied by other neurodevelopmental or emotional behavior disorders, with the most common co-morbidities encompassing attention deficit hyperactivity disorder (ADHD), autism spectrum disorder (ASD) and obsessive-compulsive disorder (OCD) (11, 12). Children with TS or other persistent TD can experience co-occurring disorders, functional impairments, discrimination, and bullying victimization or perpetration (13, 14). However, the etiology and pathogenesis of TD are currently unknown.

An increasing number of studies indicate that allergic diseases could significantly impact TD. The presence of elevated serum IgE levels and a positive skin prick test in children with TD implies that the symptoms experienced by TD may bear resemblance to allergies or be connected to allergic diseases (15, 16). Furthermore, the primary indications of TD in children are blinking of the eyes, shrugging of the nose, clearing of the throat, coughing, and may experience discomfort in their eyes and nasal passages (17). Consequently, TD can be erroneously identified as AR, AC, or AA. Studies have indicated that certain children with TD may experience an amelioration of their symptoms following antiallergic therapy (18, 19). This suggests that allergic diseases are closely related to TD in terms of pathophysiology and immunity.

This review collected studies about the correlation between allergic diseases and TD, analyzed the common factors affecting the occurrence of both, and discussed the potential mechanisms through which allergic diseases may impact TD.

## 2 Methods

In order to discuss the relationship between allergic diseases and tic disorders in children, we undertook a systematized literature search that included clinical studies and experimental studies. Searchers were conducted using Pubmed, Google Scholar, EBSCO, Scopus and Medline with the following key terms: allergy OR allergic rhinitis OR allergic conjunctivitis OR asthma OR atopic dermatitis OR food allergy AND tic disorders AND children, allergy OR tic disorders AND immunology OR neurotransmitter agents OR genetics OR gut microbiota OR sleep OR environment, allergy OR tic disorders AND mechanism OR immunological OR DNA methylation. Studies from all years were included. Some review articles and their reference lists were also searched to identify related articles.

## 3 Epidemiology

Over the past 20 years, the prevalence of childhood allergic diseases has been increasing globally (20, 21), already affecting about 25% of children (22). In 2010, the occurrence of FA among children in the US stood at 8.0% (23), while in 2019, the prevalence of FA among children aged 2 years and below in Wenzhou, China, escalated to 11.1% (24). Approximately 13% of children in the US and 15%–38% of children under the age of 5 globally are affected by AD (25). In 2013, the occurrence of AR among 12–15 years old in Europe ranged from 15.1% to 37.8% (26). From 2015 to 2017, the prevalence of AR among children in inland areas of China was 26.6%–28.5% (27, 28). A population-based study indicated that AC alone has been estimated in 6%–30% of the general population and up to 30% in children alone or association with AR (29). The number of asthma cases in the US rose from 7.3% in 2001 to 7.9% in 2017 (30), and the total number of asthma cases among Chinese children under 14 years old rose from 1.97% in 2000 to 3.02% in 2010 (31, 32). Allergic diseases recurrence rate is high, which brings great pain to and imposes a severe financial burden on patients.

At the same time, the prevalence of TD has been on the rise in the last few years (33). According to a systematic review and meta-analysis of 13 studies of children, the prevalence of TS is estimated to be 0.77%, while the prevalence of PTD is estimated to be 2.99% (34), and TD is more common in boys than girls, with a ratio between 2 and 1 and 3.5 to 1 (35). A CDC study using parent-reported data found that 1 out of every 333 (0.3%) children 3–17 years of age in the US have received a diagnosis of TS (36). The combined prevalence of TS and other TD is estimated to be over 10 cases per 1,000 (1%, 1:100), suggesting that over ½ million children have a TD in the US. And the overall rate of TD in Chinese kids varies from 1.04% to 2.98% (37, 38). This variability in rates across different regions and populations could indicate a complex interplay of genetic, environmental, and possibly cultural factors influencing the manifestation of these disorders.

## 4 Correlation between allergic and TD

In 1984, in an overview of the clinical experience of 300 TS patients, Bruun noted that “although there is no evidence of allergy as a cause of TS, my clinical experience suggests that exacerbation of TS symptoms is usually associated with seasonal allergic reactions or the ingestion of allergens in food” (39). In 1985, Finegold first reported elevated serum IgE and positive skin prick test in four patients with TS who presented to an allergy specialist. He noted that the symptoms of patients with TS can resemble allergies or appear in combination with allergic diseases (34). Mandell's letter in response to Finegold's report, said his survey of 26 TS patients showed that 80% had allergies. Unfortunately, Mandell's report does not mention any details of the materials and methods used, or the laboratory tests performed on TS patients (40). In 1987, Comings et al. after comparing 247 patients with TS and 47 with the control group, concluded that there was no statistical difference between the two groups in terms of comorbid allergic disease (41). In 1997, Kim suggested that some foods may be involved in TS because they promote the production of certain neurotransmitters (42). In 1999, a study published by Ho showed that 41 of 72 TS patients (56.9%) had clinical allergies and tested positive for allergens, which was much higher than the 44.3% allergy rate in the local population. Therefore, this study concluded that the incidence of allergy in TS patients was significantly higher than that in the general population (43). Despite the conflicting results of the aforementioned studies, there is an increasing focus on the potential link between allergic conditions and the emergence of TD.

In 2011, Chang included 845 TS patients aged 2–18 years and 3,378 controls matched to the case group in terms of age, sex, and urbanization levels based on the Taiwan Health Database, to assess the correlation between allergic diseases and TS. The results showed a significant correlation between allergic diseases and TS, with subjects with AR having a doubled risk of TS (corrected OR = 2.18), and the corrected ORs for AA, AD, and AC were 1.82, 1.61, and 1.33, respectively, and the risk of TS rises with the number of comorbid allergic conditions (44). In 2013, Chen found a significantly increased risk of TD in patients with ADHD combined with allergies, following a further expansion of the study population, according to Taiwan Health Database (45). In 2014, a preliminary study in Turkey showed a higher rate of AR diagnosis in TD patients than in controls (40.6% vs. 17.1%, *p *= 0.033), suggesting a significant correlation between allergic diseases and TD (46). In 2019, Chen reported the association between AC and TD through a case-control study and found that children with TD had a significantly higher incidence of AC (74.3% vs. 17.1%, *p* < 0.001), higher skin prick test positivity than controls (80.0% vs. 20.0%), and a history of AR was significantly associated with TD (47). It can be seen that as research progresses, more and more studies show that children with TD are more likely to be diagnosed with allergies than healthy children.

In 2021, Liu showed that allergic diseases increased the severity of TD childhood (48). In 2022, a meta-analysis provided strong evidence for a relationship between allergic diseases and TD: TD is positively associated with AA, AR, and AC (21). In 2022, Zhang et al. showed some concordance with TD in the grading and distribution of IgE and sIgE levels in children with allergic diseases (49). In the same year, Chang et al. used a case-control study found that allergic diseases were associated with the development of TD in children, with AC having the highest correlation with TD (OR = 4.95), followed by eczema (OR = 2.64), AR (OR = 2.64), and FA (OR = 2.50), and this study also found that more than 80% of the children's allergic diseases preceded the development of TD (50).

The above studies have shown that not only is there a correlation between allergic diseases and TD, but that allergic diseases may be an important risk factor for the occurrence and severity of TD. Hence, it is crucial to analyze the factors that may contribute to the simultaneous occurrence of both and delve deeper into the mechanisms through which allergic diseases impact TD, to effectively prevent and treat TD.

## 5 Common risk factors for allergy and TD

Research from various studies had highlighted common factors such as neurotransmitter imbalance, maternal anxiety or depression, gut microbial disorders, sleep disturbances, maternal allergic status, exposure to tobacco and environmental factors in both allergic diseases and TD. In the following segment, we provide proof regarding the role of these elements in allergic diseases and TD.

### 5.1 Neurotransmitter imbalance

Allergy and TD, both are governed by neurotransmitters—allergies by histamine and TD by serotonin and dopamine. Allergies can affect the nervous system, leading to symptoms such as red itchy eyes, sneezing, and altered gastrointestinal motility, which are mediated by the release of mediators that interact with sensory nerves and alter transmission in autonomic nerves (51).

Mediators linked to allergies can engage with sensory nerves, modify the central nervous system's (CNS) processing, and alter transmission in autonomic and enteric nerves, leading to neuronally-based symptoms of allergy. Synaptic interactions between presynaptic and postsynaptic components within the sympathetic, parasympathetic, and enteric ganglia are crucial for the autonomic and enteric nervous systems. Presynaptic neurons originating from the CNS are responsible for regulating efferent neurons in the majority of organs. Stimulation of the preganglionic nerve occurs in the CNS, where action potentials travel along the preganglionic axon, eventually establishing synapses with autonomic ganglia neurons. Rather than mere relay stations, these ganglia serve as locations for filtering and assimilating CNS inputs. In allergies, this could be significant as mast cells are frequently linked with ganglia that are sympathetic, parasympathetic, and enteric (51). Specifically within the intestinal tract, there exists a self-governing efferent regulation that operates independently from the CNS's neural processing. Here, a sensory nerve, upon sensing a local environmental stimulus, can relay this data straight to adjacent efferent enteric neurons via local afferent-efferent synapses. Known as a local “peripheral reflex”, these enteric ganglion neurons engage in communication via intraganglionic pathways. Other internal organs, like the airways and gall bladder, may also exhibit peripheral reflexes, though not as extensively as those in the gastrointestinal system (52).

Histamine is pivotal in the development of various allergic conditions like AD, AR, and AA, through differential regulation of T helper lymphocytes. Histamine is responsible for boosting the release of Th2 cytokines like IL-5, IL-4, IL-10, and IL-13, and suppressing the production of Th1 cytokines including IFN-γ, IL-12, and IL-2. Consequently, histamine plays a role in maintaining the equilibrium between Th1 and Th2 cells, aiding in the transition towards Th2 cells (53). Primarily found in mast cells and basophils, histamine plays a key role in allergic conditions, facilitating asthma's initial two primary symptoms (bronchospasm and edema) via its H1 receptor and mucus secretion through its H2 receptor (54). Therefore, histamine plays a crucial role in the pathophysiology of allergic diseases. Studies have revealed a critical role of the Gamma-Aminobutyric Acid (GABA) signaling pathway in the airway epithelium of AA through its ability to stimulate mucus production (55). Additionally, GABA can inhibit allergic reactions in the airways of guinea pigs by stimulating GABA receptors (56). GABA can also suppress the immune system, thereby easing the allergic responses (55). At the same time, there is evidence to suggest a relationship between serotonin, also known as 5-hydroxytryptamine (5-HT), and allergies. Allergic sensitization can modify the pulmonary expression of 5-HT receptors in guinea pigs (57). Supplementation with 5-hydroxytryptophan (5-HTP), a precursor to 5-HT, has been found to inhibit allergic lung inflammation and airway responsiveness induced by allergens (58). It can be seen that changes in various neurotransmitters are closely related to the development of allergies.

Although the exact cellular and molecular base of TS is as yet elusive, research in neuroanatomy and neurophysiology suggests the participation of cortical-striatal-thalamocortical pathways, connecting distinct areas of the frontal cortex with subcortical formations (59). Within these circuits, the transmission of messages is regulated through various neurotransmitters, including dopamine, histamine, GABA, and 5-HT (60).

While dopamine is the most regularly detected neurotransmitter alteration in TD, there's a strong probability that patients exhibit dysfunction in various neurotransmitter systems. Changes in the brain's histamine modulation system are recognized as a factor in the emergence of tics and TS, with genetic studies showing that histamine dysregulation can lead to TD. Histamine H3 receptor activation in the dorsal striatum has been found to trigger stereotypies in a mouse model of TD (61). Mice with a knockout of the histidine decarboxylase gene, which is involved in histamine production, demonstrate recurrent behavioral disorders, imbalanced dopamine levels, and modified indicators of cell function and internal signaling within the striatum (61). A study also found that individuals with TS exhibit increased GABA in brain areas linked to the planning and selection of movements (62). Another study found that GABA is involved in the pathophysiology of TD, and increased GABA levels may contribute to enhanced control over motor excitability in TS (63). In addition, 5-HT and 5-hydroxyindoleacetic acid levels may play a role in the genesis of TD, but these findings have no significant correlations with the severity of TD (64). In summary, neurotransmitter imbalance has been implicated in the development of TD, but the exact mechanisms involved are still not fully understood.

The above analyses showed that alterations in neurotransmitters, particularly histamine, GABA and 5-HT, all have an impact on allergic disease and TD.

### 5.2 Maternal anxiety or depression

Epidemiological research indicates that the health of a mother, both before conception and throughout pregnancy and after birth, play a significant influence in her child's health. The correlation between allergies and TD may manifest before birth. There is growing evidence that the presence of stress, depression, or anxiety in mothers before, during, and following pregnancy can significantly impact their offspring, rendering them more vulnerable to allergy (65–67) and/or TD (68).

A longitudinal study in Mexico, examining 601 pairs of mothers and infants, in order to comprehend the correlation between maternal depressive symptoms and childhood asthma. The study found that maternal postpartum depression or recurrent depression was highly associated with asthma in children at 48 months (66). A large data survey in Korea found that maternal depression was significantly associated with childhood asthma (OR = 2.03) and AD (OR = 1.76) (69). Schoolchildren in China were found to have a higher likelihood of developing rhinitis if their mothers had symptoms of depression during and after pregnancy (70). A cohort study from Singapore showed that elevated scores of maternal depression before conception and during pregnancy were associated with a greater chance of the offspring developing wheezing within the initial 18 months of life (71). A meta-analysis also showed a positive association between maternal depression or anxiety and asthma in offspring (72). In yet another study—a meta-analysis comprising 30 research investigations and a separate cross-sectional study involving 3,758 pairs of Italian mothers and children—it was found that prenatal maternal distress was linked to a higher likelihood of the offspring developing eczema, rhinitis and asthma (73, 74). Maternal depression or anxiety can impact the immune system of the offspring by influencing the hypothalamic-pituitary-adrenal (HPA) axis. This axis is crucial in regulating the body's immune responses to stressors. When mothers experience depression or anxiety, it can trigger increased production and release of cortisol. This reduces the expression of 11β-hydroxysteroid dehydrogenase 2 in the placenta, leading to higher levels of cortisol exposure in the fetus (75–77). An increase in cortisol levels in infants can lead to an imbalance in the HPA axis, which can activate a T-helper 2 (Th2) immune response by blocking interleukin-12, a Th1 cytokine (78), and raising Th2 levels (79, 80), thus exacerbating inflammation and causing IgE-mediated allergic reactions (67).

A British prospective cohort study investigated 14,541 pregnant women and their children and found that increased odds of TS/CTD in children at age 13 were significantly associated with maternal chronic anxiety (OR = 2.17) and antenatal depression (OR = 1.86) (81). In a separate study, it was found that a maternal background of non-specific psychiatric disorders—encompassing anxiety disorders and depressive disorders—was associated with higher odds of children experiencing TS/CTD during their childhood and adolescence (82). In addition, the severity of TS is also significantly related to maternal stress during pregnancy (83). Reduced placental monoamine oxidase A levels in maternal with depression can lead to increased 5-HT levels, which detrimental to fetal brain development (84). In addition, some studies have confirmed that regional connections in fetal brain function related to arousal and consciousness increase as maternal anxiety levels increase (85), and this pattern may be closely related to the occurrence of TD.

### 5.3 Gut microbial disorders

Gut microbial disturbance is another common factor between allergies and TD. In the past decade, many studies have been conducted to determine that microbial disturbance has an important impact on the occurrence of allergic diseases, and in TD patients, the role of microbial ecology has also received increasing attention.

A cross-sectional study of 1,440 children showed that altered gut microbial diversity is associated with reduced AD risk (86). A Japanese study shows that children with a higher abundance of *Bacteroidetes* in their gut microbiota during infancy are more likely to develop allergic diseases after the age of 2 years (87). A cohort study in Ecuador showed that increased fecal abundance of *Streptococcus* and *Bacteroidetes* and decreased abundance of *Bifidobacterium* and *Ruminococcus* in children as young as 3 months old was associated with allergy and wheezing at age 5 years (88). Research by Zhang et al. showed that the diversity of gut microbial in children with AR was reduced and the abundance of *Bacteroidetes* was significantly increased, and changes in the gut microbial were correlated with clinical symptoms (89). The gut microbiota plays a crucial role in the immune system by influencing the development of either responsive or tolerant reactions to various antigens. It achieves this by maintaining a balance between the activities of Th1 and Th2 cells while also regulating Th17 and T regulatory (Treg) cells specifically within the lamina propria (90). Alterations in gut microbial levels or diversity (dysbiosis) can disrupt mucosal immunological tolerance, leading to allergic diseases (91, 92).

The gut microbiota of children with untreated TD has higher abundance of *Bacteroides plebeius* and *Ruminococcus lactaris*, and lower abundance of *Prevotella stercorea* and *Streptococcus lutetiensis* (93)*.* Chang et al. collected the feces of 15 TD children and 10 healthy children for 16S rRNA high-throughput sequencing. They observed that compared with healthy children, the relative abundance of *Firmicutes*, *Agathobacter*, *Subdoligranulum*, *Ruminococcus_1* and *Roseburia* in TD children was decreased, while the relative abundance of *Bacteroidetes* and *Erysipelatoclostridium* increased (88). After 1 and 2 courses of acupuncture, the symptom of TD children was lower than those before treatment and the relative abundance of *Firmicutes* was decreased, while *Bacteroidetes* and *Erysipelatoclostridium* was increased. The above two studies showed that the gut microbial of TD children may be disordered, and the improvement of gut microbial is related to the relief of tic symptoms.

The common feature of children with allergies and TD is increased levels of *Bacteroidetes*. Increased levels of *Bacteroidetes* can lead to changes in short-chain fatty acid levels. Changes in SCFAs levels may be an important factor in the co-occurrence of the two.

### 5.4 Sleep disorders

Getting proper sleep is incredibly important for growth, development, and overall well-being. Healthy sleep involves getting the right amount of sleep suitable for one's age, ensuring high sleep quality, uninterrupted sleep, and the absence of sleep disorders. Sufficient sleep significantly contributes to brain development, learning, memory consolidation, emotional regulation, executive function, and various other critical functions (94). Sleep disorders encompass a broad spectrum of sleep-related issues. These can range from not getting enough sleep, experiencing trouble falling asleep, waking up too early, having low-quality sleep, facing disruptions in circadian rhythms, dealing with insomnia, to disorders related to breathing during sleep (95). Sleep disturbances can significantly affect the body's immune system, emotional state, and cognitive abilities (96).

Increasingly, there is mounting evidence indicating a correlation between sleep disorders and outcomes related to allergies. Research suggests that individuals who had insufficient sleep (≤6 h per night) had a 1.27 times higher probability of developing allergic sensitization compared to those who had adequate sleep (7–8 h per night) (97). The correlation between sleep and allergic skin conditions is particularly noteworthy in a clinical setting, as sleep disturbances can contribute to the development of allergic skin diseases (98, 99) and is positively correlated with the severity of AD (100). According to a recent study involving over 5,000 patients from 10 European countries, inadequate sleep duration (<6 h) was linked to respiratory and nasal symptoms (101). Moreover, sleep problems have been found to mediate the association between asthma and allergic rhinitis with psychological distress in children (102). A higher proportion of eosinophils in peripheral blood is associated with a more serious sleep disorder (103). Explain that allergies can also affect sleep, leading to issues such as insomnia, trouble falling asleep, trouble staying asleep, increased snoring, increased risk for sleep apnea, poor sleep efficiency, and short sleep duration (104).

In addition, sleep disorders are not uncommon in TD patients. Approximately 65% of children with TD have exhibited various forms of sleep disturbances. These issues can encompass challenges in initiating sleep and increased disruptions during sleep, leading to decreased sleep efficiency (105). A large US population-based survey showed that poor sleep quality is related to the severity of tics (106), and that proper sleep management can help reduce the intensity and effect of TD on life (107). Sleep disturbances in children with TS include increased night waking, parasomnias, and sleep onset delayed (108). The severity of TS symptoms experienced during the day has shown a significant positive correlation with the frequency of awakenings and changes in sleep stages during the night. Conversely, it has exhibited a negative correlation with sleep efficiency, indicating that more severe TS symptoms during waking hours often relate to increased disruptions in sleep and reduced overall sleep quality (109). The behavioral and cognitive impairments present in some TD patients may be related to sleep disturbances (110). Additionally, it has been reported that motor tics, associated with TS, can persist even during sleep (105). Research has indicated that TD patients combined with AR are more likely to develop sleep disorders (111). Therefore, sleep disorders may be an important factor in the co-occurrence of allergic diseases and TD.

### 5.5 Mother's allergies

Family history of allergic diseases is directly related to the occurrence and severity of allergies in offspring, especially in mothers. If the mother has allergies, the risk of the child developing allergies is approximately 45% (112). Maternal total IgE levels correlated with elevated infant IgE levels and infant eczema (113). A cohort study showed that the offspring of mothers with a history of AA and AR during pregnancy were at higher risk of developing AD and AR (20). Studies have shown that the levels of IL-10, TGF-β and Helios-induced Tregs in the cord blood of mothers with allergies all decrease, which means that the immune tolerance of such babies at the systemic level is weakened after birth, which may be caused by maternal allergies, an important mechanism for increased allergy risk in children (114).

Similarly, maternal allergies may also affect the occurrence and development of TD in children. A nationwide prospective cohort study conducted in Denmark revealed that 110 out of 2,442 children with TS had mothers with AD prior to pregnancy, indicating a correlation between maternal AD and an elevated likelihood of TS in the offspring (115). Although there is currently few studies related to maternal allergies and childhood TD, population-based cohort studies have shown that maternal asthma is significantly associated with the development of ASD and ADHD in offspring (116–118). ASD and ADHD are the most common comorbidities of TD in children, especially ADHD, which has a similar etiology and pathogenesis to TD (119).

### 5.6 Exposure to tobacco

Exposure to tobacco is a prevalent environmental factor. Extensive evidence indicates that tobacco exposure during early life doesn't just impact prenatal development but can also cause structural and functional changes. These alterations heighten the risks of immune, cardiovascular, and neuroendocrine disorders in offspring (120).

Allergic disease in children has been linked to exposure to environmental tobacco during pregnancy and early life (121). A comprehensive analysis of 43 studies encompassing 29 distinct birth cohorts revealed that smoking during pregnancy heightened the likelihood of wheezing in children under the age of 6 by 36% (OR, 1.36; 95% CI: 1.19–1.55), while also elevating the risk of asthma in children aged 6 and above by 22% (OR, 1.22; 95% CI: 1.03–1.44) (122). The meta-analysis revealed that maternal smoking during pregnancy may elevate the likelihood of recurring wheezing in infancy (123). Consequently, it can be inferred that exposure to environmental tobacco smoke elevates the risk of wheezing or asthma in children by a minimum of 20% (124). Maternal smoking during pregnancy could potentially lead to a 13% increase in the risk of AR in offspring, particularly if the mother is a passive smoker (125). Smoking while pregnant and exposure to tobacco smoke during childhood might represent non-allergic factors linked to higher risk and increased occurrence of persistent asthma or rhinitis (126). In Ferrini's study, findings indicated that prenatal exposure to tobacco makes offspring more prone to heightened allergic airway inflammation. This condition was linked to a decrease in the functioning of pulmonary NK cells (127). Exposure to tobacco can trigger an increase in the expression of Toll-like receptors (TLRs) and alter the responses mediated by lipopolysaccharides. This exposure influences the activation of nuclear factor-kB, prompts the release of IL-8 by innate lymphoid cell-2 through epithelial cytokine production, and affects chemotactic activity toward neutrophils (128). Maternal smoking during pregnancy leads to epigenetic changes, including the increased expression of miRNA-233 and a reduction in the number of Treg cells in the offspring's cord blood at birth. These changes can contribute to an inclination towards atopic conditions and an increased risk that persists during early infancy (121). Exposure to tobacco during pregnancy is considered a potential factor contributing to the development of childhood allergic diseases and this connection is believed to occur through epigenetic mechanisms (125).

A study found that maternal smoking during pregnancy and a heightened risk for TS and CTD in offspring (129). A prospective study about the Danish National Birth Cohort, which analyzed 73,073 singleton pregnancies, concluded that heavy maternal prenatal smoking was associated with an increased risk and the severity of TS/CTD in offspring (130). Additionally, a meta-analysis shows that maternal smoking during pregnancy may be associated with a 35% increase in the risk of TS and CTD among offspring (131). These findings indicate a potential association between maternal smoking during pregnancy and the development of TS and CTD in offspring. Certainly, prenatal exposure to nicotine impacts various facets of fetal brain development, influencing processes such as neuronal migration, proliferation, and differentiation. These effects can have lasting implications for the maturation and functioning of the developing brain (132). Exposure of the fetal brain to nicotine disrupts the development of various neurotransmitter systems, notably affecting dopamine, which is consistently found to be altered in studies related to TD (133, 134). Indeed, prenatal exposure to smoking can induce subtle structural changes in the brain, specifically affecting regions like the striatum, thalamus, thalamocortical fibers, and cortex (135). Abnormalities within these brain circuits have been strongly associated with the underlying mechanisms of TD, as well as their frequently occurring comorbidities such as OCD, ADHD, and ASD. Dysfunction within these circuits often correlates with the manifestation of these conditions and their interconnected nature (130). Indeed, immune dysregulation has been associated with the risk of TS/CT. Maternal smoking, known to influence immune responses, might impact fetal brain development through this mechanism, potentially contributing to the risk of these neurodevelopmental conditions.

### 5.7 Environmental pollution

Particulate matter with an aerodynamic diameter of less than 2.5 μm (PM2.5) represents a criteria pollutant known for its ability to harm the alveoli within the respiratory system. These tiny particles can penetrate blood vessels after affecting the respiratory system (136). Epidemiological studies have revealed that exposure to PM2.5 can lead to various adverse health effects in humans. These effects include increased risks of allergies, congenital heart defects, and the induction of negative impacts on neurodevelopment (137). Infants and young children are particularly vulnerable to the detrimental effects of air pollution. This susceptibility is due to the relatively immature state of their immune and respiratory systems, making them more prone to experiencing severe health impacts from exposure to pollutants like PM2.5 (138). Furthermore, children tend to spend more time outdoors and breathe in about 50% more air per kilogram of body weight compared to adults. This higher respiration rate and outdoor activity expose them to relatively higher doses of ambient pollutants, further increasing their vulnerability to the adverse effects of air pollution (139).

PM2.5 has been found to have a strong correlation with allergies and allergic respiratory diseases (140). Exposure to PM2.5 can trigger allergic reactions and worsen existing allergic conditions (141). Among them, short- and long-term exposure to ambient PM2.5 could increase the risk of allergic nasal and eye symptoms, worsening dyspnea caused by allergens, and an increase in overall allergic symptoms (142). Elevated concentrations of PM2.5 can disrupt the balance of T helper cells. High levels of PM2.5 tend to increase the expression of TNF-α and cytokines associated with Th2 responses, such as IL-4 and IL-10. Conversely, they decrease the expression of the Th1-associated cytokine IFN-γ. This imbalance alters the Th1/Th2 ratio, which plays a critical role in immune responses (143). In a study conducted on rats with AR, the researchers noted that the expression levels of IFN-γ, IL-4, IL-5, IL-33, intercellular adhesion molecule 1 (ICAM1), and vascular cell adhesion molecule 1 (VCAM1) increased in correlation with the concentration of PM2.5. This suggested higher concentrations of PM2.5 led to increased expression levels of these markers associated with AR (144). Following exposure to PM2.5 can trigger a range of pathological alterations in the lungs, especially notable in asthmatic mice. These changes often include inflammatory cell infiltration, thickening of bronchial smooth muscles, and injury to the bronchial mucosa. These effects illustrate the impact of PM2.5 on exacerbating asthma-related symptoms and lung pathology (145). The above studies have shown that PM2.5 plays a role in promoting the occurrence and development of allergic diseases.

Prenatal and postnatal exposure to PM2.5 is associated with an increased risk of TD in infants delivered at term. A study conducted in central Taiwan found that exposure to PM2.5 during pregnancy and infancy was positively associated with the risk of TD, with a vulnerable time window for infants at 6–52 weeks after birth. The hazard ratio (HR) of TD was positively associated with a 10 μg/m3 increase in PM2.5 during pregnancy (HR 1.09, 95% CI 1.04, 1.15) and infancy (HR 1.12, 95% CI 1.06, 1.18). Indeed, the study found a non-linear relationship between exposure to PM2.5 and the risk of TD. Specifically, exposure levels between 16 and 64 μg/m^3^ of PM2.5 were associated with an increased risk of TD, particularly notable within the TS group. This non-linear association suggests a specific range of PM2.5 exposure that might significantly impact the risk of developing TD (146).

**Figure 1:**
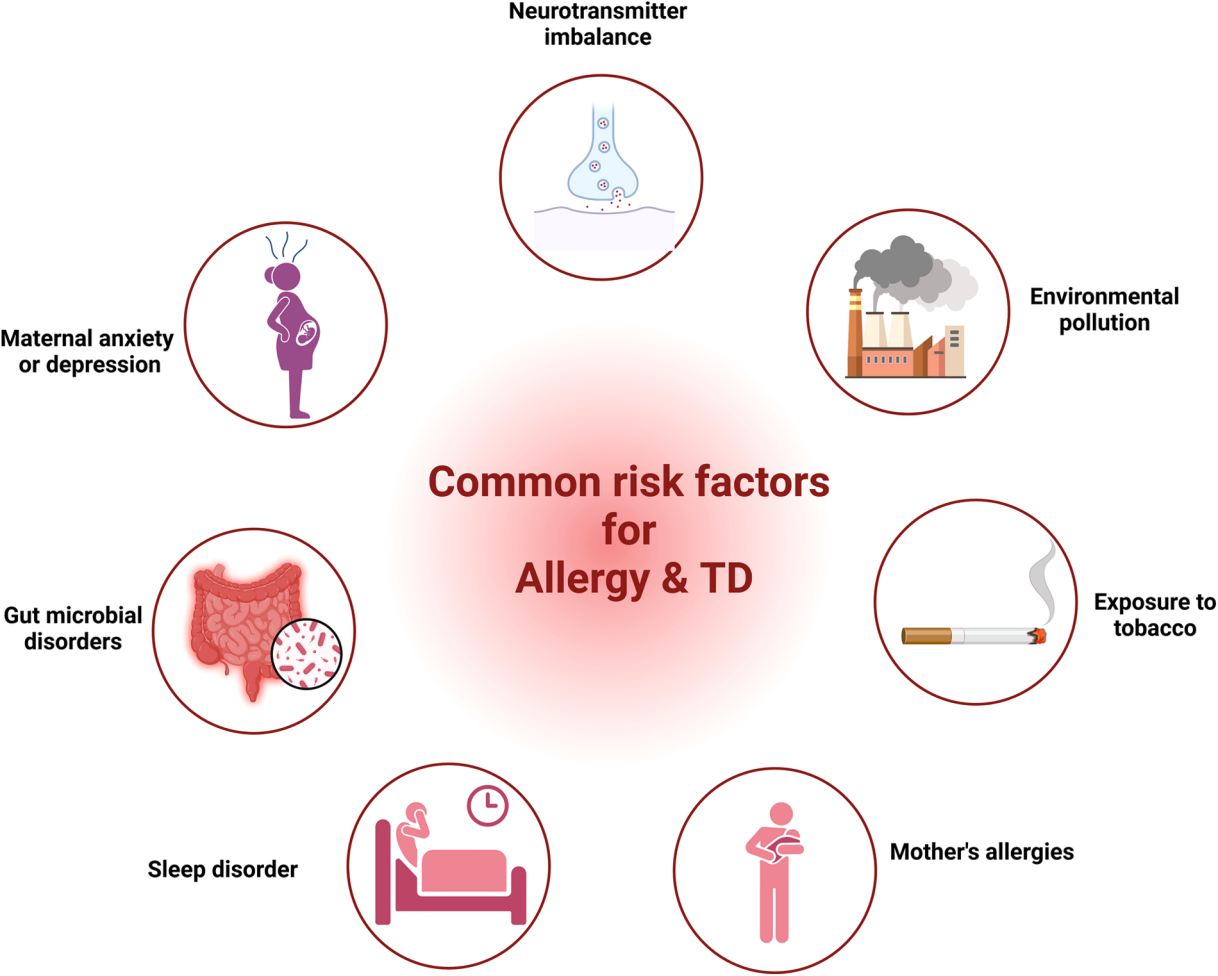
The common risk factors for allergy and TD. (Pictured by Biorender).

Our discussion covered shared elements leading to allergies and TD, encompassing neurotransmitter imbalance, maternal anxiety or depression, gut microbial disorders, sleep disturbances, maternal allergic status, exposure to tobacco, and environmental factors (Figure 1). Nonetheless, direct proof is absent to establish a link between these elements and both allergy and TD. Consequently, extended longitudinal research is essential to investigate the link between these factors and both conditions within the same group, as well as to explore how these factors mediate the relationship between allergies and TD.

## 6 Possible mechanisms linking allergy and TD

Emerging evidence pointing to potential links between allergies and TD underscores the importance to understanding the underlying mechanisms. Exploring mechanisms like alterations in gut microbial, changes in immunological and DNA methylation can provide valuable insights. These insights might lead to the identification of new treatment avenues and therapeutic targets for addressing both allergy-related TD.

### 6.1 Gut microbial

The ecosystem of the human gastrointestinal tract starts from the oral cavity, passes through the esophagus, stomach, small intestine, colon, and finally reaches the rectum. Its huge surface area of 150–200 m^2^ provides sufficient colonization space for microorganisms. The number of bacteria per milliliter of intestinal content is between 100,000 and 100 billion (147). Genome sequencing found that the gut microbial contains approximately 22.2 million genes, which is more than 700 times the length of the human genome (148). The gut microbial of a healthy human body is mainly composed of four bacterial phyla, namely *Bacteroidetes*, *Firmicutes*, *Proteobacteria* and *Actinobacteria*, accounting for more than 98% of all human gut microecologies (149). Gut microbial endows the host with multiple functions, including producing vitamins, absorbing calcium ions, resisting pathogens, enhancing immune function, etc. (150).

#### 6.1.1 Gut-brain axis

The gut-brain axis, which refers to the bidirectional communication between the gastrointestinal tract and the CNS, has become one of the hotspots of research in the medical field. Research has shown that not only can the brain regulate gastrointestinal function and homeostasis, but the gut can also affect people's mood, sleep, and even neurological development and repair in a variety of ways, and this two-way communication between the gut and brain is known as the gut-brain axis (151). Gut-brain axis communication is based on neural, immune, endocrine and metabolic pathways. Gut microbial can produce neurotransmitters directly or indirectly through host biosynthetic pathways, and dysbiosis can cause neurotransmitter disorders and neurodevelopmental disorders. Beneficial bacteria such as *Lactobacillus*, *Bifidobacterium,* and *Bacillus* have been found to produce a variety of neurotransmitters, including dopamine, norepinephrine, 5-HT, GABA, acetylcholine and histamine, etc. (152). The above-mentioned neurotransmitters enter the CNS through the enteric nervous system (ENS) and affect the physiological functions of the brain. Available studies have shown that *Lactobacillus* and *Bifidobacterium* affect the synthesis of acetylcholine and GABA, while the synthesis of 5-HT, dopamine and norepinephrine is affected by *Streptococcus*, *Enterococcus* and *Escherichia coli* (153). Meanwhile, the effects of gut microbial on the nervous system are partly mediated by bacterial metabolites, the best-known of which are SCFAs, which can affect the development of the nervous system, immune signaling, and the integrity of the blood-brain barrier (154).

The two major barriers in the signal transduction process of the gut-brain axis are the gut barrier and the blood-brain barrier, of which the gut barrier is the first barrier between the human body and the intestinal lumen, and its normal functioning affects the exchange of substances between the body and the intestinal lumen and the invasion of pathogens (155). Gut microbial increase intestinal permeability by degrading biofilms on mucosal surfaces, which translocates lipopolysaccharide-containing Gram-negative bacteria, causing overactivation of the immune system and increased levels of pro-inflammatory cytokines. Excessive release of such pro-inflammatory factors is more destructive to CNS (156). The blood-brain barrier effectively regulates the exchange between the cerebrospinal fluid and the circulatory system, keeping the internal environment of the CNS relatively stable (157). Gut microbial reduce blood-brain barrier permeability by upregulating tight junction protein expression (158). At the same time, the use of many antipsychotics, such as fluoxetine and aripiprazole, can also alter the intestinal barrier (159), leading to alterations in signaling between the brain and the gut.

#### 6.1.2 Allergy and SCFAs

The impact of allergic diseases on TD may be mediated by SCFAs, which mainly include acetic, propionic and butyric. SCFAs can influence the immune response in remote parts of the body and reduce airway inflammation caused by ovalbumin and house dust mites (160, 161). Furthermore, oral administration of SCFAs during pregnancy and weaning reduces the severity of allergic airway inflammation in offspring (162). *Bacteroidetes* and *Firmicutes* are the most common microorganisms in the intestine (163). *Bacteroidetes* are usually associated with the production of more acetic acid and propionic acid, while *Firmicutes* are associated with the production of more butyric acid (164). Butyrate is the most important presence in maintaining colon health. It can directly serve as an energy source for colon epithelial cells, maintain intestinal barrier integrity by regulating tight junction expression, and has systemic anti-inflammatory properties (165). Children with higher fecal propionic and butyric acid levels at 1 year of age are less likely to develop AA at 3–6 years of age (162). SCFAs are not only related to the integrity of the intestinal barrier, but also affect the ENS, promote the normal development of microglia, and affect the immune function and signaling of the CNS (166).

#### 6.1.3 SCFAs and gut-brain axis

The ENS is also called the “Second Brain”. It is a neuron network composed of sensory neurons, motor neurons, interneurons and supporting cells embedded in the entire gastrointestinal wall. It is connected to the CNS through the vagus nerve, forming a gut-brain signal axis (167). Studies have shown that antibiotics induce a reduction in the ENS neuron network in mice (168). Gut function and enteric neurons restored after administration of SCFAs to germ-free mice (169). This shows that SCFAs deficiency can lead to the loss of intestinal neurons, thereby weakening ENS function. Damage to the ENS can produce pro-inflammatory factors that damage tight junction proteins and change the integrity of the blood-brain barrier, thereby affecting the maturation of microglia (170, 171). Microglia are resident macrophages in the brain's innate immune system and account for approximately 10% of nervous system cells. They are critical for the clearance of pathogens, senescent cells, and synaptic remodeling during development (172). Studies have shown that germ-free mice and antibiotic-treated mice have defects in microglial maturation, activation and differentiation, and morphological changes (173). Excessive activation of microglia function can trigger a cascade of neuroinflammation, including increased levels of IL-6, IFN-γ, TNF-α, and reactive oxygen species and reactive nitrogen species, leading to damage to the blood-brain barrier and neuronal cell death and brain damage (174). The addition of SCFAs (containing butyric acid, acetic acid, and propionic acid) to germ-free mouse feed improves microglia morphology and maturation defects (173). Sodium butyrate mediates neuroprotection by downregulating pro-inflammatory mediators TNF-α and NOS_2_ and upregulating IL-10 expression in microglia (175). Butyrate can also exert anti-inflammatory effects by limiting the formation of TGF-β_1_ and IL-6, enhancing Tregs cell activity and host immunity (176). SCFAs increase the expression of the transcription factor FOXP3 by inhibiting histone deacetylation, thereby expanding Tregs (177). At the same time, SCFAs can regulate genes encoding cAMP response element-binding proteins, regulate the synthesis of catecholamine neurotransmitters (such as dopamine), and affect gut-brain axis signal transmission (178).

#### 6.1.4 SCFAs and TD

**Figure 2:**
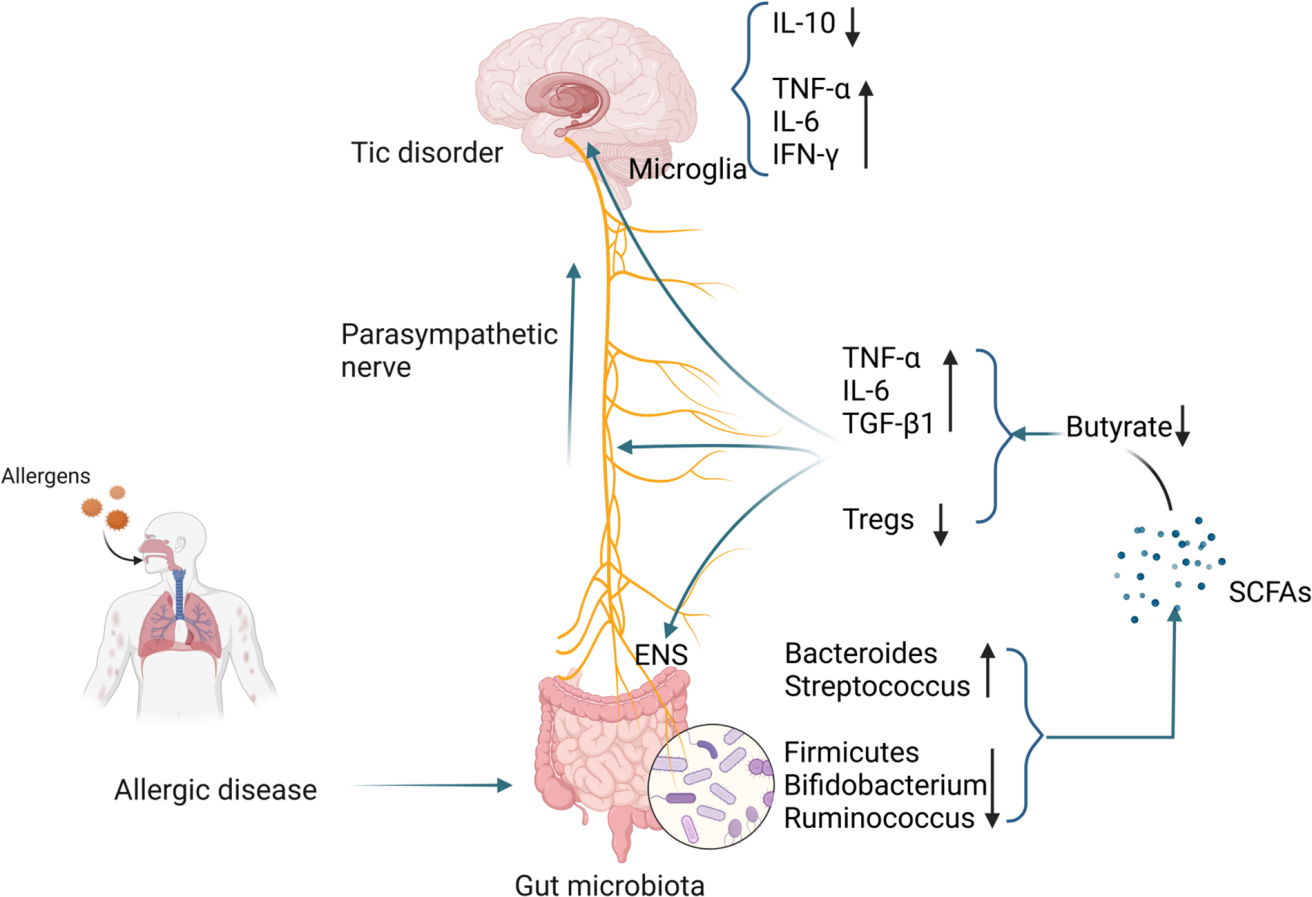
Possible mechanisms by which gut microbiota alterations occurring in patients with allergic diseases influence the development of TD. (Pictured by Biorender).

The number of microglia in the striatum of TD patients is increased, accompanied by enriched expression of inflammatory genes (179). Using PET imaging found microglia activation in the bilateral caudate nuclei of TD children and suggested that activated microglia mediate neuroinflammation (180). In animal models, it was also found that microglial activation in the striatum of TD mice was enhanced, accompanied by increased levels of pro-inflammatory cytokines IL-1β and TNF-α. The above studies all point to the activation of microglia in TD (181). Compared with healthy controls, the levels of pro-inflammatory mediators such as TNF-α, IL-6, IL-1β, and IL-12 in the plasma of TD children were significantly higher (182). Other studies have shown that excessive activation of immune responses in children with TD may be caused by decreased levels of peripheral Tregs (183). A case-control study showed that the concentration of tetrahydroisoquinoline in the urine of TD children was significantly increased. Tetrahydroisoquinoline can regulate dopaminergic neurotransmission and metabolism in the CNS, indicating that dopaminergic overactivity exists in TD children (184). Changes in SCFAs in patients with allergic diseases, especially the decrease in butyrate levels, may damage the ENS, activate microglia, and trigger a neuroinflammation cascade, resulting in decreased Tregs levels and dopaminergic overactivity, thereby inducing the occurrence of TD. The specific mechanism is shown in Figure 2.

### 6.2 Changes in immunological

#### 6.2.1 Allergy and immunity

The occurrence of allergic diseases is related to immune imbalance mediated by T lymphocytes. When a child is exposed to an allergen for the first time, dendritic cells (DC) process it and present it to CD4+ T cells. CD4+ T cells are activated and differentiate into Th2-type cells, producing IL-4 and IL-5. and IL-13 (185). IL-4 and IL-13 induce the activation of B cells and antibody class switching to produce sIgE (186). sIgE reaches various parts of the body through the circulation system and interacts with mast cells and basophils. The IgE high-affinity receptor (Fc*ε*RⅠ) on the cell surface binds, putting the body in a sensitized state (187). When the child is exposed to the same allergen again, the allergen binds to sIgE on the surface of mast cells and basophils, sIgE cross-links with Fc*ε*RⅠ, and mast cells and basophils degranulate, leading to histamine, etc. (188–190). IL-5 promotes the production and maturation of eosinophils, and the major basic protein released by IL-13 stimulates mast cells to release histamine and leukotrienes to trigger allergic symptoms (191).

Recent studies have also shown that epithelial cells are also involved in the development of allergies. Epithelial cells are the first line of defense against environmental damage and can produce a variety of cytokines after exposure to allergens, such as IL-25, IL-33, and thymic stromal lymphopoietin. These cytokines promote Th2 differentiation through DC and type 2 innate lymphocytes (192). In addition, reduced levels of Tregs were also observed in the peripheral blood of patients with allergic diseases (193). Animal studies have shown that infusion of Tregs from normal mice into ovalbumin-induced allergic mice significantly inhibited their airway hyperresponsiveness, while reducing eosinophil infiltration and lowering IL-5 in bronchoalveolar lavage fluid and IL-13 levels (194). Insufficient differentiation and functional defects of Tregs are key factors in the enhancement of Th2 responses and the occurrence of allergies, which are mainly manifested in the fact that TGF-β and IL-10 secreted by Tregs can significantly inhibit airway inflammation and hyperresponsiveness while blocking TGF-β or IL-10 aggravates airway inflammation and hyperresponsiveness (195). Studies have shown that the levels of TNF-α, IL-6 and other cytokines are increased in allergic diseases (196, 197). Although TNF-α is thought to synergize with IL-17 to promote neutrophil accumulation, TNF-α can also promote the production of Th2 cytokines, enhance airway smooth muscle contraction, and contribute to the occurrence of airway hyperresponsiveness (191). The above-mentioned studies have shown that patients with allergic diseases have significant immune system imbalances.

#### 6.2.2 Immunity and TD

Increasing evidence shows that imbalances in the immune system also play an important role in the development of neurological diseases (198). Pro-inflammatory cytokines are released by the activation of immune cells when the host responds to pathogen invasion, tissue damage, and psychosocial stress. During an immune attack, pro-inflammatory cytokines are generally released for a short period and are regulated by anti-inflammatory mechanisms. Therefore, the immune signal released by the CNS in response to inflammation is an adaptive, temporary, and controllable response. However, when the immune attack becomes chronic and/or unregulated, the behavioral effects of cytokines and the resulting inflammatory response may promote the occurrence of TD (199, 200). Some studies have shown that the occurrence or worsening of tic symptoms in TD patients is related to abnormal immune activation caused by infection, and some TD patients have increased serum anti-streptolysin levels (201). Mycoplasma and enterovirus infections are also associated with tic severity (202). Therefore, when children with allergic diseases are repeatedly exposed to allergens, the immune imbalance caused by them and the continuous attack of pro-inflammatory cytokines may be important mechanisms in inducing TD.

#### 6.2.3 Immune relationship between allergy and TD

**Figure 3:**
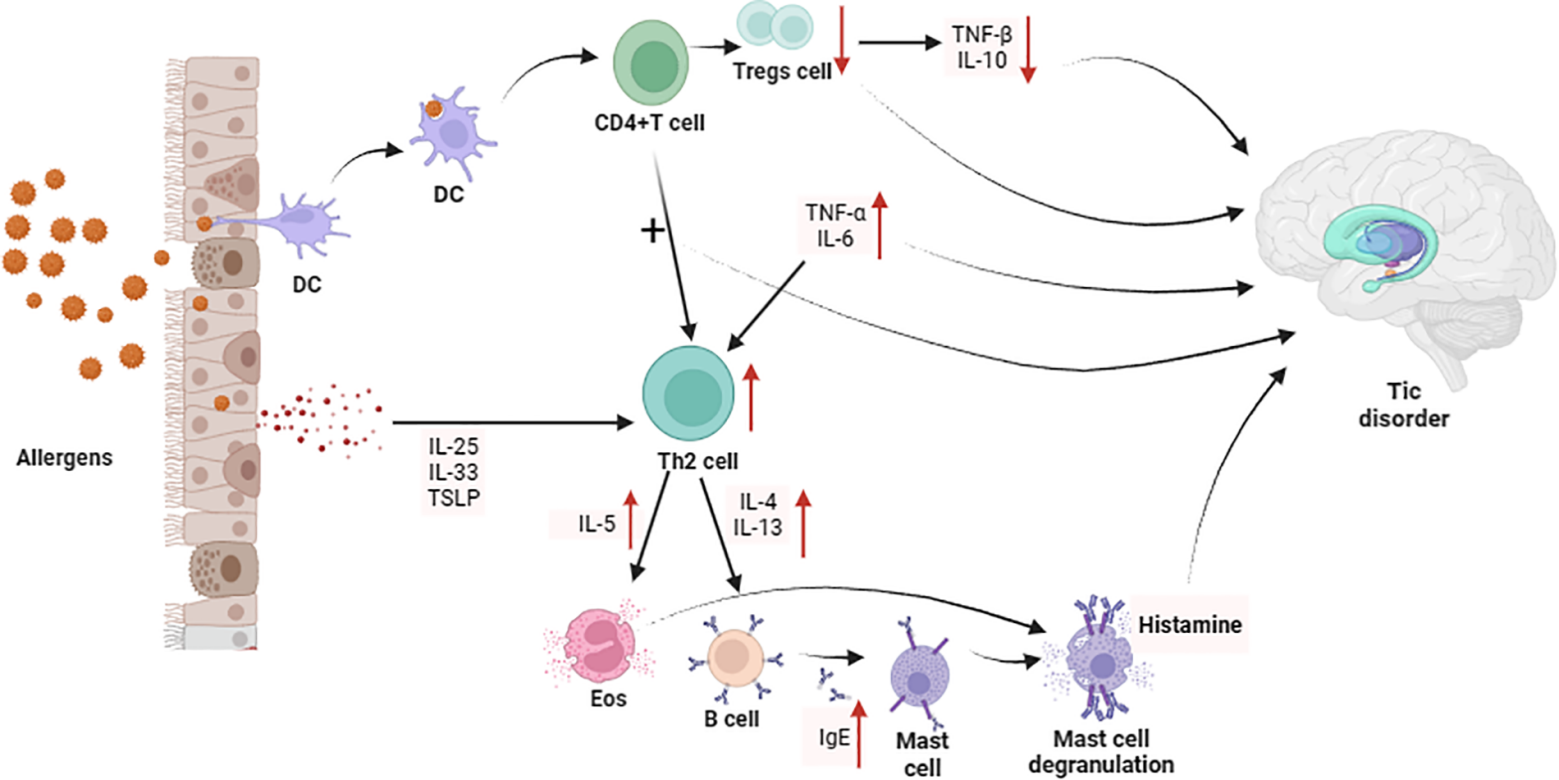
Possible mechanisms by which immunological changes occurring in patients with allergic diseases influence the development of TD. (Pictured by Biorender).

Allergic diseases will aggravate the imbalance of T lymphocyte subsets and impairment of cellular immune function in TD children, mainly manifested by the decrease in the expression levels of CD3+ T cells, CD4+ T cells, and CD4+/CD8+ (48). Leckman found that the levels of pro-inflammatory cytokines such as TNF-α and IL-12 were increased in the peripheral blood of children with TD, and during the worsening of symptoms, the levels of these two cytokines further increased, suggesting that the occurrence of TD is related to innate immunity. Related to T cell immune imbalance (203). Another study showed that levels of pro-inflammatory cytokines such as IL-17A, IL-6, IL-12, and TNF-α were elevated in TD children without obsessive-compulsive disorder (202). Compared with healthy controls, TD children who did not receive drug treatment had higher TNF-α levels in the peripheral blood (204). Animal studies have shown that IL-6 increases 5-HT and dopamine activity in the hippocampus and frontal cortex of mice, showing more frequent digging, rearing, and grooming (205). Injection of IL-6 into mice during mid-pregnancy can lead to a lack of prepulse inhibition in offspring, and TD patients often have abnormal performance in this sensorimotor process (206). In addition, a study showed that the number of Tregs cells decreased in patients with moderate to severe TD, and further decreased during the symptom exacerbation period (183). This phenomenon may be related to the decrease in Tregs cells caused by the inflammatory response caused by allergies and the reactive pathogenic phenotype of Tregs (202). Histamine is an important mediator in the pathogenesis of allergic diseases, and its receptor-mediated signaling pathways are significantly enriched in TD patients, further indicating that the occurrence of allergic diseases may be an important risk factor for TD. In addition, some TD patients do not respond well to antipsychotic drugs, but they do respond well to adrenocorticotropic hormone or plasma exchange (37, 38). The above results all suggest that immune imbalance in allergic diseases and the resulting changes in related cytokine levels may be important factors in inducing TD. The specific mechanism is shown in Figure 3.

### 6.3 DNA methylation

The increasing prevalence of allergic diseases in children indicates that environmental exposures such as microbial imbalance, air pollution, and pet ownership have an important impact (207). Environmental factors can have persistent effects on gene expression by regulating epigenetic DNA methylation (208). DNA methylation refers to the transfer of methyl groups to cytosine-guanine (CpG) dinucleotides using S-adenosylmethionine as the methyl donor under the action of DNA methyltransferase. On the fifth carbon atom of cytosine. DNA methylation plays a pivotal role in modulating gene expression by changes in chromatin structure, DNA conformation, DNA stability, and the interactions between DNA and proteins. These modifications serve as a mechanism for controlling which genes are expressed or silenced within a cell (209).

#### 6.3.1 Allergy and DNA methylation

At present, many studies have shown that DNA methylation testing in patients with allergic diseases may be helpful in the differential diagnosis of the disease (20). A large-scale cross-sectional study collected whole blood from 392 asthmatic children aged 4–8 years old and 1,156 control children for a whole-epigenome association study. This study discovered 14 differentially methylated CpG sites, which were subsequently verified through a meta-analysis of six additional European cohort studies (4–16 years old, 247 children with asthma and 2,949 controls). This study showed that all 14 differentially methylated CpG sites were significantly associated with asthma. These CpG sites are associated with whole blood transcriptional profiles, which are associated with increased activation of eosinophils, CD8+ T cells, NK cells, and reduced numbers of naive T cells (210). Another study showed that the DNA methylation of CpG sites in blood monocytes and airway epithelial cells of children with allergic diseases changed (211). After sublingual immunotherapy in patients with respiratory allergies, the CpG methylation level of the FOXP3 site in Tregs cells was reduced (95). In addition, studies have shown that DNA hypermethylation can cause a decrease in IFN-γ levels in AR patients, and this change may help distinguish allergic patients from healthy people (212). At the same time, DNA methylation in allergic diseases may also affect neurodevelopment, especially inducing the occurrence and development of TD.

#### 6.3.2 TD and DNA methylation

A German study showed that compared with healthy people, the methylation level of the dopamine D2 receptor gene in TD patients was significantly increased and was positively correlated with the severity of tics. The dopamine transporter methylation level is negatively correlated with tic severity (213). Another epigenome-wide association study on TD investigated differences in DNA methylation in Dutch twins with TD. Among the top-ranked probes, an enrichment of differentially methylated neural genes previously associated with neurological diseases was detected (214).

#### 6.3.3 DNA methylation link between allergy and TD

Studies have shown that compared with controls, patients with allergic diseases have differentially methylated CpG sites on Cadherin-26 (CDH26) (215). As an important cell adhesion molecule, CDH26 mediates cell-cell, cell-extracellular matrix interactions (216–218), and can regulate leukocyte migration, adhesion and activation, especially in the context of allergic inflammation (219). Tsetsos et al. also found in a genome-wide association study that CDH26 is related to TD and contains four single nucleotide polymorphisms (SNPs) (220). SNPs are the most common form of human genetic variation and represent changes in a single base pair in an individual's DNA sequence. Many SNPs are known to be associated with various human diseases, among which the genetic variations associated with TD are mainly concentrated in dopamine receptors (221). Dopamine receptors also play an important role in the occurrence of allergic diseases in children. Their combination with dopamine promotes Th2 cell differentiation and enhances Th2 inflammation in mice (222). At present, there are few epigenetic related studies on TD. Based on the analysis of existing research data, DNA methylation in allergic diseases, especially the differentially methylated CpG sites in CDH26, may be related to the occurrence of TD, but the relationship between DNA methylation between the two still needs to be further explored.

## 7 Conclusion

In summary, this study believes that there is a clear relationship between allergic diseases and TD, and allergic diseases may be an important risk factor for the occurrence of TD. Therefore, early and active treatment of allergic diseases may effectively prevent the occurrence of TD. There are currently few studies on the relationship between allergic diseases and TD, especially the lack of large-scale prospective cohort studies to further verify the causal relationship between the two. At the same time, it is of great significance to conduct in-depth research on the related mechanisms of allergic diseases affecting TD, aiming to provide new ideas and new directions for the prevention and treatment of TD.
